# In vivo assessment of neuroinflammation in progressive multiple sclerosis: a proof of concept study with [^18^F]DPA714 PET

**DOI:** 10.1186/s12974-018-1352-9

**Published:** 2018-11-13

**Authors:** Marloes H. J. Hagens, Sandeep V. Golla, Martijn T. Wijburg, Maqsood Yaqub, Dennis Heijtel, Martijn D. Steenwijk, Patrick Schober, John J. P. Brevé, Robert C. Schuit, Tristan A. Reekie, Michael Kassiou, Anne-Marie van Dam, Albert D. Windhorst, Joep Killestein, Frederik Barkhof, Bart N. M. van Berckel, Adriaan A. Lammertsma

**Affiliations:** 1 VUmc MS Center Amsterdam, VU University Medical Center, De Boelelaan 1117, 1081 HV Amsterdam, the Netherlands; 2 Department of Neurology, VU University Medical Center, De Boelelaan 1117, 1081 HV Amsterdam, the Netherlands; 3 Department of Radiology and Nuclear Medicine, VU University Medical Center Amsterdam, De Boelelaan 1117, 1081 HV Amsterdam, the Netherlands; 4 Philips Healthcare, Best, the Netherlands, Veenpluis 4, 5684 PC Best, the Netherlands; 5 Department of Anatomy and Neurosciences, VU University Medical Center Amsterdam, De Boelelaan 1117, 1081 HV Amsterdam, the Netherlands; 6 Department of Anaesthesiology, VU University Medical Center Amsterdam, De Boelelaan 1117, 1081 HV Amsterdam, the Netherlands; 7 School of Chemistry, University of Sydney, F11, Eastern Ave, Sydney, NSW 2006 Australia; 8 Institutes of Neurology and Healthcare Engineering, UCL Institute of Neurology, Queen Square, London, WC1N 3BG UK

**Keywords:** [^18^F]DPA714, Multiple sclerosis, Neuroinflammation, Positron emission tomography, TSPO

## Abstract

**Background:**

Over the past decades, positron emission tomography (PET) imaging has become an increasingly useful research modality in the field of multiple sclerosis (MS) research, as PET can visualise molecular processes, such as neuroinflammation, in vivo. The second generation PET radioligand [^18^F]DPA714 binds with high affinity to the 18-kDa translocator-protein (TSPO), which is mainly expressed on activated microglia. The aim of this proof of concept study was to evaluate this in vivo marker of neuroinflammation in primary and secondary progressive MS.

**Methods:**

All subjects were genotyped for the rs6971 polymorphism within the TSPO gene, and low-affinity binders were excluded from participation in this study. Eight patients with progressive MS and seven age and genetic binding status matched healthy controls underwent a 60 min dynamic PET scan using [^18^F]DPA714, including both continuous on-line and manual arterial blood sampling to obtain metabolite-corrected arterial plasma input functions.

**Results:**

The optimal model for quantification of [^18^F]DPA714 kinetics was a reversible two-tissue compartment model with additional blood volume parameter. For genetic high-affinity binders, a clear increase in binding potential was observed in patients with MS compared with age-matched controls. For both high and medium affinity binders, a further increase in binding potential was observed in T2 white matter lesions compared with non-lesional white matter. Volume of distribution, however, did not differentiate patients from healthy controls, as the large non-displaceable compartment of [^18^F]DPA714 masks its relatively small specific signal.

**Conclusion:**

The TSPO radioligand [^18^F]DPA714 can reliably identify increased focal and diffuse neuroinflammation in progressive MS when using plasma input-derived binding potential, but observed differences were predominantly visible in high-affinity binders.

**Electronic supplementary material:**

The online version of this article (10.1186/s12974-018-1352-9) contains supplementary material, which is available to authorized users.

## Background

Conventional magnetic resonance imaging [1] has a central role in the diagnosis of multiple sclerosis (MS), based on its high sensitivity for identifying MS-specific demyelinating lesions in the brain and spinal cord [2]. In patients with a relapsing-remitting disease course, radiological monitoring is based on the development of new MS lesions on T2-weighted MRI and the presence of gadolinium-enhancing lesions on T1-weighted MRI, demonstrating disruption of the blood-brain barrier which is indicative of active neuroinflammation [3]. However, these focal lesions as seen on MRI cannot fully explain the neurological and cognitive deficits in patients with MS [4]. In addition, in both primary and secondary progressive MS, neuroinflammation usually is not characterised by focal active demyelinating lesions [5]. Animal model and human post-mortem studies have identified a more diffuse and low-grade neuroinflammation behind an intact blood-brain barrier, not depicted by conventional MRI [6, 7]. Consequently, there is a need for novel imaging techniques.

Over the past decades, positron emission tomography (PET) imaging has become an increasingly useful research modality in the field of MS research, as PET can visualise molecular processes, such as neuroinflammation, in vivo [8, 9]. Specifically, the 18-kDa translocator-protein (TSPO) receptor, which is present on the mitochondrial membrane of microglial cells, with additional binding sites in monocytes, astrocytes and vascular endothelium, has shown potential as a target for imaging neuroinflammation with PET [10]. Using the first-generation TSPO PET tracer *(R)*-[^11^C]PK11195, increased TSPO expression has been demonstrated in both MS lesions and normal appearing brain tissue of patients with MS, which was associated with increased disability and disease progression in all phases of the disease [11–16]. Unfortunately, *(R)*-[^11^C]PK11195 suffers from poor brain penetration and high nonspecific binding in the brain, hindering accurate quantification [12]. Over the years, a large number of second-generation TSPO tracers have been developed, such as [^11^C]PBR28, [^18^F]PBR111, [^11^C]DPA713, [^18^F]GE180 and [18F]FEDAA1106. These novel tracers have an improved signal-to-noise ratio, resulting from lower lipophilicity and therefore less non-specific binding to e.g. plasma proteins and improved blood-brain barrier passage [17–21]. It has been shown that the second-generation radioligand [^18^F]DPA714 provides increased bioavailability to brain tissue and indeed has a higher signal-to-noise ratio [22, 23]. One limitation of second-generation tracers, including [^18^F]DPA714, is their genetically determined binding affinity, resulting from a single nuclide polymorphism rs6971 within the TSPO gene. Approximately 50% of the general population is a genetically determined high-affinity binder (HAB), 30–40% a medium-affinity binder (MAB) and 10–20% a low-affinity binder (LAB) [24].

To date, quantification of cerebral [^18^F]DPA714 binding has been described in healthy controls [25] and in patients with Alzheimer’s disease [26], with both studies agreeing on a reversible two-tissue compartment model with blood volume parameter (2T4k_V_B_) as the optimal kinetic model. In addition, pseudo-quantitative analyses have been performed in post-stroke [27] and Alzheimer’s disease patients [28]. Moreover, the simplified reference tissue model, using cerebellar grey matter as a reference region, has been found to reliably assess binding potential in Alzheimer’s disease [26]. However, the pathophysiology of MS violates the assumptions underlying reference tissue models: firstly, no single brain region can be used as a reference region in MS, as no region can be assumed to be devoid of specific TSPO binding, and secondly, the blood volume contribution is not negligible throughout the MS brain due to potential disruption of the blood-brain barrier [29]. The latter will also influence the kinetics of the tracer making reference tissue models unreliable [30].

The aim of this proof of concept study was to identify the optimal plasma input tracer kinetic model for characterising in vivo [^18^F]DPA714 kinetics in patients with primary and secondary progressive MS and to evaluate whether the kinetic parameters estimated from this model can be used to quantify TSPO binding in this patient group.

## Methods

### Participants

Patients diagnosed with primary or secondary progressive MS, according to the 2010 revisions of the McDonald criteria, and healthy controls were recruited from the MS Center, VU University Medical Center, Amsterdam, between January 2015 and February 2016. All subjects were genotyped for the rs6971 polymorphism within the TSPO gene, which predicts binding affinity to the second generation TSPO tracers [24]. Only genetically high and medium affinity binders (HAB and MAB) were included, and genetically low-affinity binders (LAB) were excluded. Further exclusion criteria for all subjects were a medical history of relevant neurological or auto-immune disease (other than MS); known cardiac, haematological, oncological or renal diseases; current or previous alcohol or drug abuse; recent treatment with immunomodulating drugs; or the use of benzodiazepines. None of the patients with progressive MS were on disease-modifying therapy, and no patients were treated with intravenous methylprednisolone for the last 3 months prior to the PET scans. All subjects had normal physical examination and all healthy controls a normal neurological examination.

This study was approved by the Medical Ethics Review Board of the VU University Medical Center and was registered under number 2014.356. All subjects gave written informed consent prior to participation.

### MRI acquisition

MR imaging was performed on a 3 Tesla Ingenuity TF PET-MR system (Philips Medical Systems, Best, The Netherlands) on the same day as the PET-CT acquisition. MRI analysis included 3D T1 (repetition time 7.9 ms, echo time 4.5 ms, flip angle 8°, measured voxel size 1 × 1 × 1 mm^3^) for region of interest (ROI) definition and 3D T2 Fluid Attenuated Inversion Recovery (FLAIR) (repetition time 4800 ms, echo time 279 ms, inversion time 1650 ms, measured voxel size 0.9 × 0.9 × 1.1 mm^3^) for lesion segmentation. In patients, additional post-contrast 2D T1 sequences (repetition time 600 ms, echo time 10 ms, measured voxel size 0.56 × 0.44 × 5 mm^3^) were acquired.

### PET acquisition and reconstruction

Radiosynthesis of [^18^F]DPA714 was performed with an in-house built automatic apparatus using procedures described previously [26]. PET scans were acquired on an Ingenuity TF PET-CT scanner (Philips Medical Systems, Best, The Netherlands). Following a low-dose CT scan for attenuation correction, a bolus of 263 ± 13 MBq [^18^F]DPA714, with a mean molar activity of 39 ± 20 GBq/μmol, was injected intravenously at 0.8 mL/sec with an automated infusion pump. Simultaneously, a 60-min PET dynamic emission scan was started. The emission scan was collected in list mode and rebinned into 19 frames (1 × 15, 3 × 5, 3 × 10, 4 × 60, 2 × 150, 2 × 300, 4 × 600 s). PET data were reconstructed to a final voxel size of 2 × 2 × 2 mm^3^ using standard scanner software (BLOB-OS-TF), which incorporates standard corrections for attenuation, scatter and randoms.

Arterial blood was withdrawn continuously from the radial artery during the entire scan (using an automated pump for the first 5 min at 5 mL/min and from 5 to 60 min at 2.5 mL/min), and in addition, manual samples were collected at six time points (5, 10, 20, 30, 40 and 60 min) [31]. Continuous whole blood time activity curves (TACs) were corrected for plasma to whole blood ratios, metabolites and time delay to obtain metabolite-corrected arterial plasma input functions.

### Data analysis

MS white matter lesions were automatically segmented on the FLAIR and T1-weighted images using kNN-TTP [32]. Lesion filling of the T1-weighted image was obtained using LEAP [33], after which the filled T1-weighted image was registered to the PET scan using the VINCI software package [34, 35]. Grey matter, white matter and cerebral spinal fluid segmentation was performed automatically using SPM8 implemented in PVElab [36]. ROIs were defined according to the Hammers template on the co-registered 3D T1 MRI [37]. Regional TACs were extracted by superimposing the MR-derived ROIs onto the dynamic PET images. Volume weighted larger ROIs were also defined to assess the impact of ROI size on quantitative analyses. The lesion masks were subtracted from segmented white matter to define non-lesional white matter.

Kinetic modelling was performed using standard single-tissue (1T2k) together with reversible and irreversible two-tissue (2T3k and 2T4k) compartment models, both with and without blood volume parameter (V_B_) [38]. It has been hypothesised that some binding to the vascular endothelium exists for TSPO tracers, which can be characterised by slow irreversible binding (K_b_) [39, 40]. Therefore, a 2T4k_V_B_ with an irreversible vascular binding component (1T1k) was also tested. Akaike information criterion (AIC) was used to compare model fits and to identify the optimal tracer kinetic model. Reliability of parameter estimates was determined by the percentage standard deviation (%SD) of these estimates, with a cut-off of 25% for the generally robust K_1_, and 50% for the other parameters. Estimates for K_1_, BP_ND_ (k_3_/k_4_), *V*_T_ and K_b_ from the optimal model were used to assess group differences between patients and controls, and between genetically high and medium affinity binders (HAB and MAB).

### Statistical analysis

Group differences were evaluated using the Mann-Whitney *U* test in SPSS 22.0 (IBM Corp., Armonk, NY). Due to the explorative nature of this proof of concept study, we did not correct for multiple comparisons. To evaluate regional differences within the patient group, a Wilcoxon signed-rank test was used.

## Results

### Demographics

At screening, all subjects were genotyped for the rs6971 polymorphism within the TSPO gene and low-affinity binders were excluded from participation in this study. In total, eight patients diagnosed with primary or secondary progressive MS and seven healthy controls completed the whole study protocol. Demographic information is reported in Table 1. Patients included were four HAB and four MAB, with a mean age of 53 ± 3 years and a wide variation in T2 lesion load, ranging from a few solitary lesions to widespread confluent lesions (3.6 to 60.6 cm^2^). In only two patients, one small enhancing lesion could be identified, not uncommon in primary or secondary progressive MS. In healthy controls, genetic binding status was similarly distributed: three HAB and four MAB. The mean age for healthy controls was 52 ± 4 years, which was not significantly different from the patients with MS (*p* = 0.418).

**Table 1:** Demographical characteristics

	Patients (*n* = 8)	Healthy controls (*n* = 7)
Age, mean and SD (years)	53.1 ± 2.7	52.0 ± 4.1
Gender, male/female	3/5	3/4
Genotype, HAB/MAB	4/4	3/4
Subtype MS, PPMS/SPMS	5/3	N/A
Disease duration, mean and SD (years)	13.1 ± 9.4	N/A
EDSS, median and range	5.0 (4.0–6.0)	N/A
PASAT-3 score, mean and SD	45.8 ± 10.8	49.6 ± 12.5
SDMT score, mean and SD	44.0 ± 11.9	55.0 ± 13.5
T2 lesion volume, median and range (cm^3^)	14.3 (3.6–60.6)	N/A

*Abbreviations*: *EDSS* Expanded Disability Status Scale, *HAB* genetic high-affinity binder, *MAB* genetic medium-affinity binder, *PASAT* Paced Auditory Serial Addition Test, *PPMS* primary progressive multiple sclerosis, *SDMT* Symbol Digit Modalities Test, *SPMS* secondary progressive multiple sclerosis

### Tracer kinetics

**Fig. 1:**
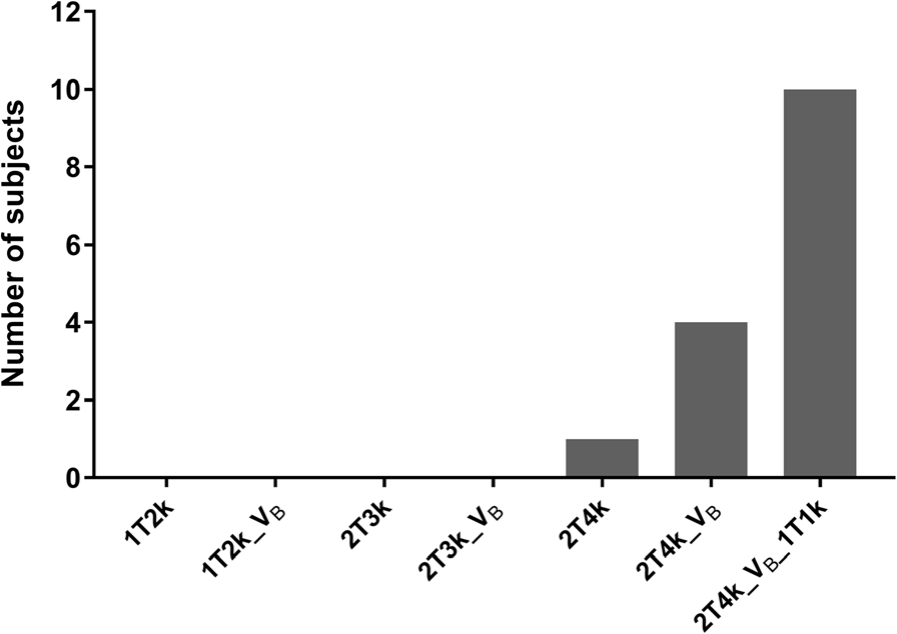
Model preference. Number of subjects per preferred model according to the Akaike information criterion. All subjects preferred a reversible two-tissue compartment model (2T4k), primarily for the model including blood volume parameter [38] and a vascular binding component (1T1k)

**Fig. 2:**
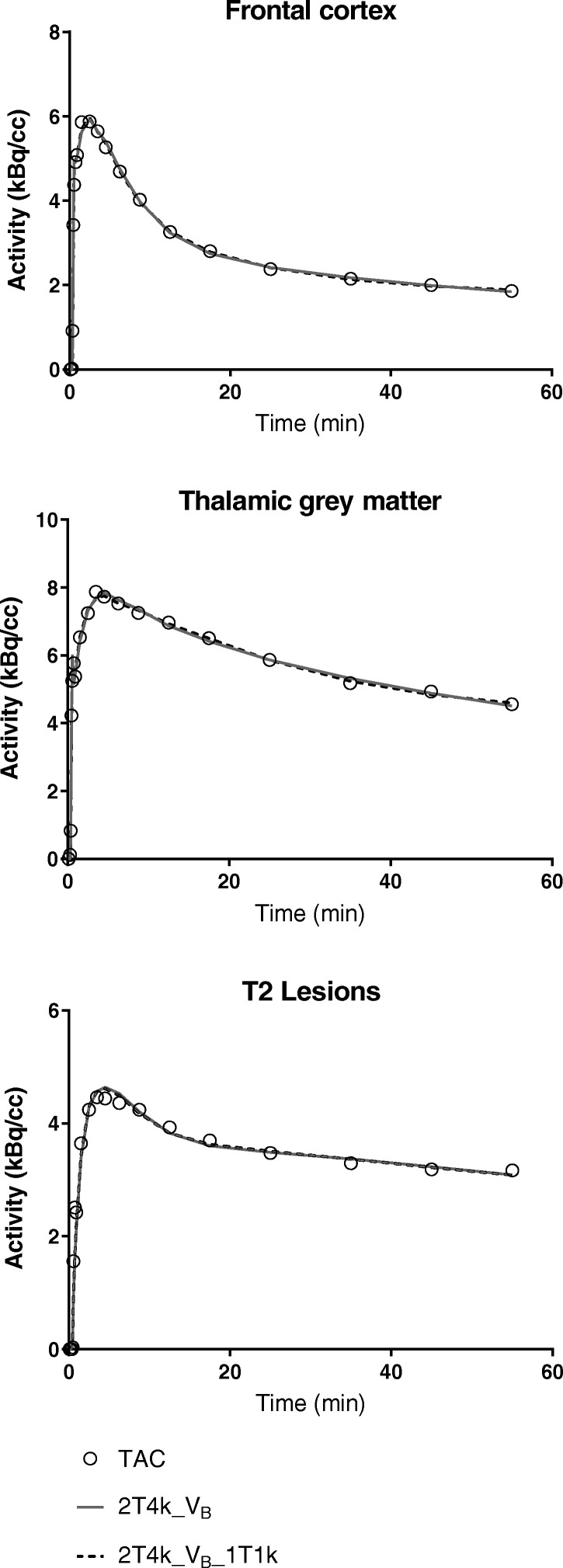
Model fit comparisons. Representative model fits for the frontal cortex of a medium affinity binding healthy control, the thalamic grey matter of a high-affinity binding MS patient and the T2 lesions of a medium-affinity binding MS patient. The open circles represent the measured time-activity curves (TAC) and the solid grey and dashed black lines represent the fits for the reversible two-tissue compartment model without and with vascular binding component (2T4k_V_B_ and 2T4k_V_B__1T1k)

Based on the AIC, for the majority of subjects, the preferred model for quantification of [^18^F]DPA714 was 2T4k_V_B__1T1k, see Fig. 1. However, when evaluating reliability of the parameter estimates, for a large number of regions, K_1_, BP_ND_ and/or K_b_ could not be reliably estimated, with either a high %SD or values touching the boundaries (see Additional files 1 and 2). This was even the case for subjects in which 2T4k_V_B__1T1k was the preferred model according to AIC, and it was true not only for small, but also for large ROIs. As model fits for 2T4k_V_B__1T1k and 2T4k_V_B_ were comparable (see Fig. 2) and 2T4k_V_B_ did result in reliable parameter estimates, the latter model was used for further analyses.

#### Volume of distribution

**Fig. 3:**
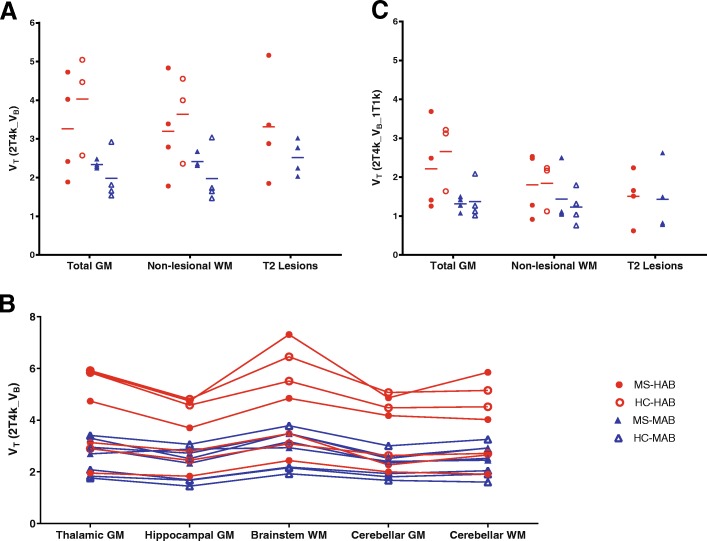
Volume of distribution. **a** Volume of distribution (*V*_T_) for total grey matter non-lesional white matter (WM) and T2 defined white matter lesions for the reversible two-tissue compartment model (2T4k_V_B_). High-affinity binding (HAB) subjects in red have a 1.5 to 2-fold higher *V*_T_ than medium affinity binding (MAB) subjects in blue. No difference in *V*_T_ was seen between multiple sclerosis patients (MS; closed symbols) and healthy controls (HC; open symbols) for both HABs and MABs. **b** *V*_T_ across various regions of all patients and controls. The intra-subject variation appears to be much smaller than the inter-subject variation. **c** The reversible two-tissue compartment model with vascular binding component (2T4k_V_B__1T1k) also identified an increase in *V*_T_ for HAB subjects compared with MAB subjects, but no difference in mean regional *V*_T_ for patients compared to controls for either HABs or MABs

**Fig. 4:**
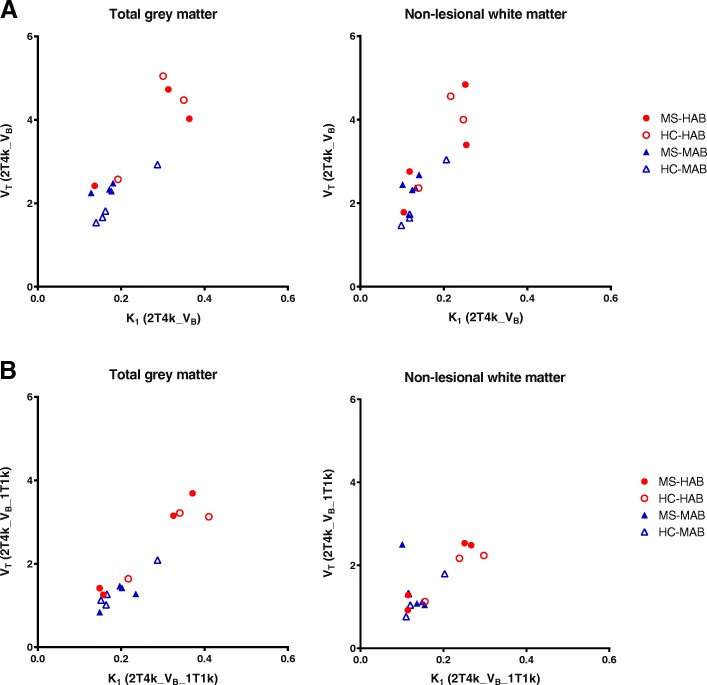
Correlation for *V*_T_ and K_1_. For both the reversible two-tissue compartment model **a** without and **b** with vascular binding component (2T4k_V_B_ and 2T4k_V_B__1T1k), there was a strong correlation between *V*_T_ and *K*_1_ in both grey and white matter, independent of binding status

Mean regional *V*_T_ values were on average 1.6 times higher in HABs than in MABs (Fig. 3a). There was no clear regional difference in *V*_T_ between patients and healthy controls for both HABs and MABs, as Fig. 3a illustrates for the MS white matter lesions, non-lesional white matter and whole brain grey matter. Moreover, very limited within-subject variation in *V*_T_ was observed, even for the patients with MS. Figure 3b illustrates that all subjects showed a similar distribution pattern for *V*_T_ throughout the brain. In addition, a strong correlation between *V*_T_ and K_1_ was seen (Fig. 4a), whereas no correlations were found between *V*_T_ and both k_2_ and k_3_/k_4_.

Finally, regional *V*_T_ values for the 2T4k_V_B__1T1k model were evaluated (Fig. 3c). As expected, the added vascular binding component decreased the *V*_T_. As the regional *V*_T_ values for the two models were strongly correlated for the large ROIs, there was also no difference in 2T4k_V_B__1T1k *V*_T_ between patients and healthy controls for both the HABs and MABs (Fig. 3c). It is of interest to know that also the correlation between *V*_T_ and K_1_ as seen for the simpler model remained the same for this model incorporating a vascular binding component (Fig. 4b).

#### Binding potential

**Fig. 5:**
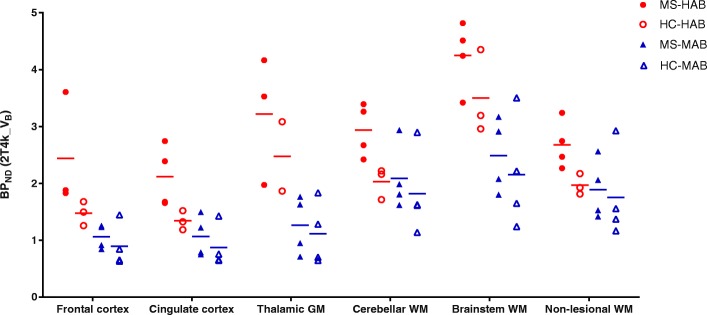
Binding potential for different regions of interest. For high-affinity binders, a higher binding potential (BP_ND_) was seen in multiple sclerosis patients (MS-HAB) than in healthy controls (HC-HAB) for different regions of interest. This difference was not seen in medium-affinity binding (MAB) subjects

BP_ND_ (k_3_/k_4_) estimates for the larger ROIs were reliable using the 2T4k_V_B_ model, as for almost all regions, the standard deviation was well below 50% for both HABs and MABs (in both patients and controls), see Additional file 3. This indicates that plasma input-derived BP_ND_ can be used as outcome parameter for these larger brain regions. Like *V*_T_, regional BP_ND_ was on average 1.6 times higher in HAB than in MAB subjects (Fig. 5). For the regions shown in Fig. 5, this difference was statistically significant for frontal cortex (*p* = 0.001), cingulate cortex (*p* = 0.006), thalamic grey matter (*p* = 0.002) and brainstem white matter (*p* = 0.004). In contrast to the *V*_T_ findings, regional BP_ND_ in both grey and white matter of HAB patients was higher than in HAB healthy controls. This difference was statistically significant for cingulate cortex (*p* = 0.034), cerebellar white matter (*p* = 0.034) and non-lesional white matter (*p* = 0.034). This difference was not observed in the MAB group.

### Multiple sclerosis lesions

**Fig. 6:**
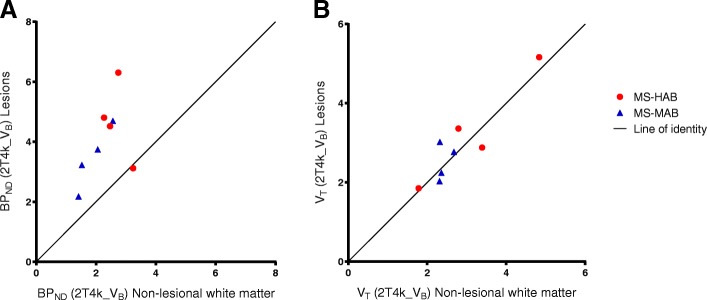
Kinetic parameter values for the multiple sclerosis lesions. Independent of binding status, **a** binding potential (BP_ND_) identified increased [^18^F]DPA714 binding in MS-specific brain lesions compared with non-lesional white matter in the same patients, **b** whereas volume of distribution (*V*_T_) did not depict this difference

In both HAB and MAB patients with MS, MRI-defined T2 white matter lesions showed a significant increase in BP_ND_ compared with non-lesional white matter of the same subject (*p* = 0.017) (Fig. 6a). There was a strong correlation between the BP_ND_ values, except for one patient. When including all the subjects, the correlation coefficient (*R*^2^) was 0.246 (slope 1.04 ± 0.744), excluding the one outlier improved the *R*^2^ to 0.861 (slope 2.39 ± 0.429). For *V*_T_, values were similar for lesions and non-lesional white matter of each MS patient, with an *R*^2^ of 0.844 and slope 1.02 ± 0.178 (Fig. 6b).

## Discussion

This proof of concept study in patients with progressive MS showed that quantitative assessment of the second-generation TSPO radiotracer [^18^F]DPA714 can identify an increased binding potential not only in MS-specific lesions, but for HAB patients also in grey matter and non-lesional white matter. Although the results from this study need to be replicated in a larger cohort, they suggest that [18F]DPA714-PET could be used as an in vivo imaging technique for measuring neuroinflammation in progressive MS.

Neuroinflammation in MS is characterised by complex and dynamic processes, including the activation of microglia [6, 7]. This is most distinct in active MS lesions, identified by gadolinium enhancement on T1-weighted MRI, and chronic MS lesions as seen on T2-weighted MRI [3]. In line with this, an increase in [^18^F]DPA714 BP_ND_ was seen in the focal lesions compared with non-lesional white matter for all patients except one, independent of the patient’s genetic binding status (HAB or MAB). As MRI-defined lesions for these patients with progressive MS were almost exclusively non-enhancing chronic lesions, this increase TSPO binding indicates that these are chronic active lesions [41]. The classification of different stages of MS lesions, such as chronic active and chronic inactive lesions, results from pathological studies. Current conventional MRI techniques can identify active inflammatory lesions, with the use of gadolinium-enhanced T1-weighted scans; however, they cannot further differentiate non-enhancing lesions. Results from the present study suggest that [^18^F]DPA714 PET can identify different stages of MS lesions in vivo, which could be clinically relevant, e.g. in relation to treatment options and monitoring.

Even more importantly, increased [^18^F]DPA714 BP_ND_ was also seen in non-lesional brain parenchyma, both in white and grey matter, for HAB patients. In relapsing-remitting MS, neuroinflammation is evident from the development of new focal (gadolinium enhancing) lesions, but in primary and secondary progressive MS, neuroinflammation is often more diffuse and low-grade [5]. Therefore, it is highly relevant that a biomarker of neuroinflammation in MS demonstrates not only focal changes, but also more diffuse pathology. As [^18^F]DPA714 BP_ND_ can identify both, this marker of TSPO expression can be used as a biomarker to further investigate neuroinflammation in MS in vivo.

As plasma input-derived BP_ND_ was estimated reliably using the 2T4k_V_B_ model, BP_ND_ should be preferred over the less specific *V*_T_. As *V*_T_ contains both free, non-specific and specific signals, it is an inferior outcome measured compared with BP_ND_ (=k_3_/k_4_), which only reflects specific binding. Contrary to BP_ND_ analysis, group analysis using *V*_T_ showed no difference between patients and controls for both HAB and MAB subjects. In addition, *V*_T_ appeared to be almost similar between different ROIs within each subject, resulting from a nearly homogeneous distribution of DPA714 throughout the brain of an individual. As *V*_T_ includes the large non-displaceable compartment of [^18^F]DPA714, this effectively obscured any changes in the disease-specific neuroinflammatory signal. This is also seen in the analysis of the T2 MS lesions in Fig. 6, illustrating that the increase in specific [^18^F]DPA714 signal in MS lesions depicted by BP_ND_ is diluted in the *V*_T_ values due to the non-displaceable component.

Interestingly, *V*_T_ was strongly correlated with K_1_. Although a change in K_1_ can be related to neuroinflammation, as K_1_ is a function of blood flow and vascular permeability, a correlation between *V*_T_ and K_1_ was unexpected as *V*_T_ should be independent of K_1_. A possible explanation for this finding could be related to binding of [^18^F]DPA714 to the endothelium of blood vessels in the brain [42, 43]. However, the model incorporating a vascular binding component still provided correlated K_1_ and *V*_T_ values (Fig. 4b).

Although the 2T4k_V_B__1T1k model was preferred according to AIC, it decreased the reliability of the parameter estimates compared with the simpler 2T4k_V_B_ model. Moreover, K_b_ estimates were inaccurate for many regions, even in subjects where this model was preferred. A proposed method of decreasing the number of parameters, attempting to increase their reliability, is fixing *V*_B_ to 5%. However, due to the large inter- and intra-subject variation in *V*_B_, this seems inappropriate.

Although we used the simpler model in our analysis, the results are similar, as the regional *V*_T_ values for the two models correlate.

In line with other TSPO studies, assessment of the rs6971 polymorphism defined binding status is important also for quantitative analysis of [^18^F]DPA714. Although observed differences between HABs and MABs were smaller than those observed for some other second-generation TSPO tracers [24], both mean *V*_T_ and BP_ND_ values were on average 1.6 times higher for HABs compared with MABs for the different brain regions. This indicates that the selection of subjects based on binding status is essential. Furthermore, group differences between HABs were only observed when using BP_ND_, suggesting that [^18^F]DPA714 in MS could best be studied in HABs only. Due to basal expression of TSPO, the contrast for [^18^F]DPA714 appears to be too low for MAB subjects to identify patients from controls. Strikingly, the increase in BP_ND_ in MS lesions was also seen in MAB patients, indicating that [^18^F]DPA714 can identify neuroinflammation in MAB subjects in MS lesions, but due to high non-specific binding of the tracer, it is not evident in other ROIs with more low-grade neuroinflammation. As only approximately half of the general population is HAB, this is a limitation of second-generation TSPO studies.

This study is limited mainly by its small sample size. Because of the limited number of subjects in each group, only limited statistical analysis was performed. Nevertheless, based on the promising results of this pilot project, further studies using a larger sample size are warranted. This would allow for evaluation of possible correlations between [^18^F]DPA714 BP_ND_ and clinical outcome scores, such as Expanded Disability Status Scale and Symbol Digit Modalities Test. Finally, assessment of a possible relationship with MRI-derived outcome measures, such as atrophy of cortical and deep grey matter structures, would be of great interest in understanding progressive MS.

## Conclusions

Plasma input-derived BP_ND_ can reliably be estimated for [^18^F]DPA714, and a 2T4k_V_B_ model [^18^F]DPA714 BP_ND_ can identify increased neuroinflammation in both grey and non-lesional white matter of patients with progressive MS, with a further increase in T2 MS lesions. As these differences cannot be seen using *V*_T_ due to inclusion of non-displaceable uptake, BP_ND_ is recommended for analysis of [^18^F]DPA714 studies in patients with MS. Similar to other second-generation TSPO tracers, the effect of genetically defined binding status limits the clinical use of [^18^F]DPA714. Nevertheless, further studies are warranted to confirm the results of the present proof of concept study.

## Additional files

Additional file 1: 2T4k_V_B__1T1k - Percentage standard deviation for BP_ND_ (k_3_/k_4_). Additional file provides the percentage of standard deviation for the for 2T4k_V_B__1T1k BP_ND_ (k_3_/k_4_) for the different large regions of interest. (PDF 106 kb)

Additional file 2: 2T4k_V_B__1T1k - Percentage standard deviation for k_b_. Additional file provides the percentage of standard deviation for the for 2T4k_V_B__1T1k k_b_ for the different large regions of interest. (PDF 106 kb)

Additional file 3: 2T4k_V_B_ - Percentage standard deviation for BP_ND_ (k_3_/k_4_). Additional file provides the percentage of standard deviation for the arterial input-derived BP_ND_ (k_3_/k_4_) for the different large regions of interest. (PDF 106 kb)

## Abbreviations

1T2k: Single-tissue compartment model
2T3k: Irreversible two-tissue compartment model
2T4k: Reversible two-tissue compartment model
BP_ND_: Binding potential (k_3_/k_4_)
FLAIR: Fluid Attenuated Inversion Recovery
HAB: High-affinity binder
k_b_: Vascular binding component
LAB: Low-affinity binder
MAB: Medium-affinity binder
MRI: Magnetic resonance imaging
MS: Multiple sclerosis
PET: Positron emission tomography
ROI: Region of interest
TAC: Time activity curve
TSPO: 18-kDa translocator-protein
*V*_B_: Blood volume parameter
*V*_T_: Volume of distribution
