# Supplementary material for: In vivo assessment of neuroinflammation in progressive multiple sclerosis: a proof of concept study with [18F]DPA714 PET
Source: J Neuroinflammation. 2018 Nov 13;15:314. doi: 10.1186/s12974-018-1352-9 (PMC6234549; doi:10.1186/s12974-018-1352-9)
Supplement: Supplementary file 1 — 2T4k_VB_1T1k - Percentage standard deviation for BPND (k3/k4). Additional file provides the percentage of standard deviation for the for 2T4k_VB_1T1k BPND (k3/k4) for the different large regions of interest. (PDF 106 kb) [file 12974_2018_1352_MOESM1_ESM.pdf]

Supplementary Table 1: 2T4k_V_B_1T1k - Percentage standard deviation for BP_{ND} (k_3/k_4)

	MS-HAB				MS-MAB				HC-HAB			HC-MAB			
	1	2	3	4	1	2	3	4	1	2	3	1	2	3	4
Frontal cortex	91.9%	18.6%	15.6%	25.1%	44.8%	25.7%	147.5%	38.6%	36.2%	24.5%	32.3%	87.7%	36.7%	22.4%	29.5%
Paracentral cortex	77.6%	14.7%	16.7%	17.8%	49.0%	73.8%	113.1%	31.1%	39.4%	25.1%	22.7%	73.0%	38.8%	17.9%	23.8%
Parietal cortex	82.2%	10.5%	16.8%	22.3%	37.3%	33.9%	90.3%	28.4%	33.7%	26.1%	24.7%	71.1%	37.4%	16.9%	28.1%
Temporal cortex	89.7%	18.0%	21.4%	30.2%	39.4%	40.3%	101.8%	42.1%	64.4%	29.0%	37.4%	89.5%	52.7%	30.1%	35.5%
Occipital cortex	90.9%	12.4%	17.9%	9.3%	51.7%	24.6%	79.9%	28.3%	23.0%	25.8%	31.1%	88.2%	18.8%	12.3%	17.0%
Cingulate cortex	96.5%	27.2%	18.8%	40.9%	34.9%	29.4%	387.5%	62.8%	70.7%	42.7%	40.5%	47.1%	57.8%	22.9%	38.5%
Thalamic GM	75.6%	21.9%	32.5%	19.0%	41.5%	40.0%	190.4%	35.4%	28.8%	39.0%	177.9%	74.4%	92.6%	31.4%	22.8%
Hippocampal GM	64.0%	38.3%	24.2%	45.1%	48.7%	22.4%	252.7%	87.9%	35.8%	29.3%	45.7%	61.0%	94.6%	63.4%	42.4%
Cerebellar GM	80.4%	13.1%	17.4%	9.6%	56.7%	30.4%	88.7%	34.6%	20.8%	29.5%	23.1%	127.8%	24.7%	13.7%	20.2%
Cerebellar WM	70.9%	14.3%	16.7%	15.8%	40.8%	22.8%	764.8%	24.8%	23.1%	17.6%	19.7%	153.8%	20.8%	14.1%	10.1%
Brainstem WM	67.9%	14.1%	20.2%	30.9%	55.0%	22.5%	306.3%	44.9%	23.6%	17.8%	23.0%	43.5%	42.5%	35.1%	15.4%
T2 MS lesions	201.8%	24.3%	27.5%	33.4%	134.4%	18.1%	599.7%	27.1%							

Abbreviations: GM = grey matter, HAB = high affinity binder, HC = healthy control, MAB = medium affinity binder, MS= multiple sclerosis, WM = white matter
