# Supplementary material for: In vivo assessment of neuroinflammation in progressive multiple sclerosis: a proof of concept study with [18F]DPA714 PET
Source: J Neuroinflammation. 2018 Nov 13;15:314. doi: 10.1186/s12974-018-1352-9 (PMC6234549; doi:10.1186/s12974-018-1352-9)
Supplement: Supplementary file 2 — 2T4k_VB_1T1k - Percentage standard deviation for kb. Additional file provides the percentage of standard deviation for the for 2T4k_VB_1T1k kb for the different large regions of interest. (PDF 106 kb) [file 12974_2018_1352_MOESM2_ESM.pdf]

Supplementary Table 2: 2T4k_V_B_1T1k - Percentage standard deviation for k_b

	MS-HAB				MS-MAB				HC-HAB			HC-MAB			
	**1**	**2**	**3**	**4**	**1**	**2**	**3**	**4**	**1**	**2**	**3**	**1**	**2**	**3**	**4**
Frontal cortex	166.4%	23.4%	49.4%	19.8%	46.4%	9.0%	25.5%	16.7%	24.2%	17.2%	21.8%	163.6%	23.0%	35.9%	11.9%
Paracentral cortex	171.1%	17.5%	133.2%	14.0%	48.7%	23.2%	23.9%	14.3%	27.1%	16.2%	20.7%	99.8%	16.6%	36.6%	10.8%
Parietal cortex	152.9%	20.1%	58.8%	14.2%	45.2%	11.5%	16.4%	11.6%	19.8%	13.7%	21.3%	82.2%	12.7%	22.2%	9.9%
Temporal cortex	150.3%	22.1%	74.4%	22.5%	50.3%	13.5%	20.5%	18.3%	40.7%	18.2%	26.7%	278.5%	31.7%	42.8%	12.5%
Occipital cortex	152.6%	11.5%	51.7%	5.3%	59.8%	9.4%	15.6%	11.5%	11.8%	12.3%	17.5%	129.7%	16.0%	26.5%	7.1%
Cingulate cortex	167.4%	49.0%	63.0%	32.7%	33.2%	12.2%	>999%	21.2%	42.1%	24.4%	41.1%	67.7%	37.1%	60.1%	15.5%
Thalamic GM	258.8%	27.2%	178.4%	18.2%	66.2%	12.2%	29.7%	15.0%	46.0%	22.4%	98.6%	160.4%	>999%	32.4%	14.9%
Hippocampal GM	141.2%	29.0%	66.4%	26.8%	55.9%	4.8%	>999%	36.3%	32.1%	29.1%	31.2%	328.6%	72.6%	91.7%	31.3%
Cerebellar GM	169.9%	18.2%	40.9%	5.6%	56.7%	48.3%	27.2%	14.8%	12.8%	14.6%	18.3%	223.7%	10.7%	18.8%	8.4%
Cerebellar WM	18.8%	18.0%	30.7%	12.5%	48.9%	17.5%	>999%	17.5%	18.0%	12.3%	18.8%	>999%	14.7%	30.5%	6.7%
Brainstem WM	21.5%	21.5%	50.1%	21.8%	42.9%	16.2%	>999%	24.9%	44.6%	18.4%	21.5%	155.9%	35.9%	27.3%	14.6%
T2 MS lesions	740.0%	27.2%	42.9%	29.2%	212.5%	16.5%	>999%	21.6%							

Abbreviations: GM = grey matter, HAB = high affinity binder, HC = healthy control, MAB = medium affinity binder, MS= multiple sclerosis, WM = white matter
