# Supplementary material for: In vivo assessment of neuroinflammation in progressive multiple sclerosis: a proof of concept study with [18F]DPA714 PET
Source: J Neuroinflammation. 2018 Nov 13;15:314. doi: 10.1186/s12974-018-1352-9 (PMC6234549; doi:10.1186/s12974-018-1352-9)
Supplement: Supplementary file 3 — 2T4k_VB - Percentage standard deviation for BPND (k3/k4). Additional file provides the percentage of standard deviation for the arterial input-derived BPND (k3/k4) for the different large regions of interest. (PDF 106 kb) [file 12974_2018_1352_MOESM3_ESM.pdf]

Supplementary Table 3: 2T4k_V_B - Percentage standard deviation for BP_{ND} (k_3/k_4)

	MS-HAB				MS-MAB				HC-HAB			HC-MAB			
	1	2	3	4	1	2	3	4	1	2	3	1	2	3	4
Frontal cortex	51.4%	14.5%	15.4%	20.0%	13.7%	11.3%	22.9%	14.4%	22.4%	21.5%	19.0%	12.7%	9.8%	9.0%	12.5%
Paracentral cortex	47.7%	12.7%	15.7%	18.7%	14.3%	17.2%	36.9%	13.7%	20.5%	21.0%	19.5%	13.7%	10.3%	7.8%	11.9%
Parietal cortex	41.8%	9.3%	16.3%	18.8%	14.2%	9.0%	15.0%	10.3%	18.1%	19.3%	16.9%	13.3%	8.4%	7.0%	10.3%
Temporal cortex	48.9%	13.0%	19.9%	20.7%	17.9%	9.9%	15.5%	14.3%	32.0%	20.0%	23.4%	15.4%	15.1%	11.9%	11.2%
Occipital cortex	39.7%	11.4%	17.6%	14.4%	16.4%	9.2%	14.5%	10.2%	14.4%	14.9%	18.2%	12.6%	6.4%	4.6%	9.4%
Cingulate cortex	51.0%	16.8%	17.3%	25.2%	11.8%	10.8%	11.6%	15.4%	33.3%	23.6%	28.3%	15.6%	25.6%	10.9%	14.6%
Thalamic GM	61.0%	13.2%	29.9%	23.0%	19.0%	9.8%	35.2%	20.8%	24.8%	30.4%	89.7%	21.4%	16.9%	11.8%	23.0%
Hippocampal GM	38.9%	21.8%	22.6%	33.7%	24.0%	10.3%	19.4%	32.6%	31.4%	33.2%	30.8%	32.2%	53.9%	38.3%	18.5%
Cerebellar GM	38.2%	9.7%	17.8%	15.7%	14.5%	17.5%	27.0%	11.6%	12.4%	17.1%	21.1%	13.5%	9.5%	6.6%	10.4%
Cerebellar WM	30.9%	11.6%	16.2%	13.8%	13.1%	14.0%	18.3%	14.9%	12.8%	16.2%	17.4%	18.4%	9.5%	7.5%	10.8%
Brainstem WM	45.5%	11.8%	18.8%	23.0%	19.6%	18.2%	14.1%	20.4%	17.8%	15.8%	20.5%	23.3%	20.6%	23.4%	15.4%
T2 MS lesions	36.3%	12.7%	20.0%	20.4%	12.6%	13.1%	18.2%	14.3%							

Abbreviations: GM = grey matter, HAB = high affinity binder, HC = healthy control, MAB = medium affinity binder, MS= multiple sclerosis, WM = white matter
